# Evaluating Adult's Competency: Application of the Competency Assessment Process

**DOI:** 10.1155/2015/753873

**Published:** 2015-07-15

**Authors:** Dominique Giroux, Sylvie Tétreault, Marie-Pier Landry

**Affiliations:** ^1^Rehabilitation Department, Faculty of Medicine, CHU de Québec Research Center, Université Laval, Québec, QC, Canada G1V 0A6; ^2^Occupational Therapy Department, Haute École de Travail Social et de la Santé, EESP, 1010 Lausanne, Switzerland; ^3^French Studies and Professional Writing program, Université Laval, Québec, QC, Canada G1V 0A6

## Abstract

Competency assessment of adults with cognitive impairment or mental illness is a complex process that can have significant consequences for their rights. Some models put forth in the scientific literature have been proposed to guide health and social service professionals with this assessment process, but none of these appear to be complete. A new model, the Competency Assessment Process (CAP), was presented and validated in other studies. This paper adds to this corpus by presenting both the CAP model and the results of a survey given to health and social service professionals on its practical application in their clinical practice. The survey was administered to 35 participants trained in assessing competency following the CAP model. The results show that 40% of participants use the CAP to guide their assessment and the majority of those who do not yet use it plan to do so in the future. A large majority of participants consider this to be a relevant model and believe that all interdisciplinary teams should use it. These results support the relevance of the CAP model. Further research is planned to continue the study of the application of CAP in healthcare facilities.

## 1. Introduction

Assessing the legal competence of adults is a complex process that entails the careful consideration of a variety of factors. One of these factors is that the results of these assessments can have a considerable impact on the person's rights [1]. In Québec, as in other provinces and in many countries, the concept of competency refers to “the level of judgment and decision-making ability needed to manage one's own affairs and to sign official documents” [2]. Competency assessment requires determining the capacity of a person to take care of himself or manage his property [1].

People with mental illness or cognitive impairments are particularly vulnerable to the risk of abuse [3]. Both they and their property need to be protected, but due consideration must be given to issues related to the loss of rights and independence that can result from setting up protective supervision [4].

Different types of legal protection measures exist for individuals who have been declared legally incompetent, but the law and the processes that lead to that declaration differ from country to country as well as from province to province in Canada. In Québec, in order to request protective supervision, medical and psychosocial assessments must first be made and submitted to the court. A judge or clerk then considers the results of the assessments submitted to determine the most appropriate type of protective supervision needed: protection measure with mandatory; protective supervision with an adviser; tutorship; or curatorship.

Regardless of the law, the tools and steps used to assess clinical competency of individuals to manage their finances and live independently remain the same. These tools should bring to light a person's ability to process information and make decisions in order to meet all of their needs and to ensure their safety. Therefore, the impact of cognitive impairment on a person's functioning capacities and the risks associated with this impairment must be well documented, as this will determine whether it is appropriate to open a protective supervision [2, 5–9]. Authors agree on the importance of using a functional model for the clinical evaluation of incapacity [8–12]. Indeed, the legitimacy of neuropsychological assessments is increasingly being challenged, and many authors insist on the relevance of evaluating not only individuals' decision-making abilities, but also their ability to act on their decisions, to adapt to life's demands, and to ensure their safety [8–11, 13].

A previous research by Giroux [14] led to the development of a clinical competency assessment model, the Competency Assessment Process (CAP). This model guides and supports health and social service professionals through the process of determining a person's need for legal protection. A Competency Assessment Tool (CAT) was derived from the CAP and was content validated by health and social service professionals (for a detailed description of the CAP, see Giroux et al. [15]). This tool details all the relevant elements that need to be documented. The objective of this paper is to present the new model, the CAP, and to report on its clinical application by interdisciplinary teams.

## 2. Context of Competency Evaluation

Several authors recommend that competency assessments must be carried out by interdisciplinary teams [5, 13, 16–19]. Health and social service professionals (physicians, occupational therapists, nurses, social workers, psychologists, neuropsychiatrists, etc.) should work together to produce a comprehensive assessment covering the different areas of expertise of each team member, thereupon reducing the risk of finding someone incompetent without due cause.

Moreover, this decision should not only be based on a fair and rigorous assessment, but it must also take into account the ethical issues involved. Thus, in addition to a collaborative approach, it is important to include the ethical considerations in the assessment process and to think about the impact a declaration of incompetence would have on the individual and family [7, 17]. Professionals must be sure to know the person being assessed (values and beliefs, environment, etc.) and base their recommendations on all of the information collected and properly documented [11, 17, 18].

Assessments must be objective, rigorous, and ethical and done with proper tools [20]. To achieve this, professionals must also be familiar with the law that defines the different legal protection measures because some are less restrictive than others [21]. It is also important to use validated tools in the assessment process. In many places, including Québec, health and social service professionals do not use a normative framework or model for decision-making; therefore, there is no standardized approach for assessing competency [18, 19, 21].

In 2003, Grisso [5] proposed a model specifically aimed at assessing competency in a legal context. It includes four assessment components (functional, causal, interactive, and judgmental), which are presented in a linear model. This model is widely reported in the scientific literature and currently the most often used. However, clinicians mention little use of this model because numerous variables derived from the four components are not detailed enough to guide decision-making [14]. A previous study highlighted the need to add additional steps to Grisso's proposed model, such as a step pertaining to risk analysis and another to ethical discussion [22]. Moreover, to ensure that clinicians use a decision-making model, it is very important to clearly describe all the underlying variables [21]. Despite its limitations, the model proposed by Grisso was considered in the development of the CAP. Steps delineated in that model Grisso were used but reorganized in a dynamic rather than linear configuration. Furthermore, steps were added to the process and underlying variables were established for each step. The following section describes in detail the proposed model: the Competency Assessment Process (CAP).

## 3. Description of the CAP and CAT

**Figure 1:**
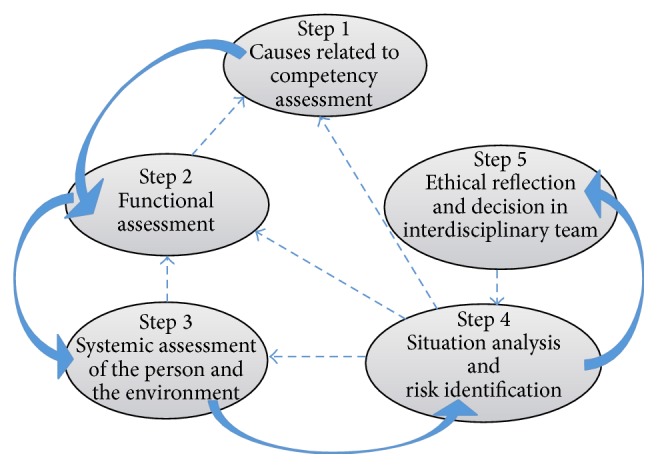
Competency Assessment Process, Giroux [14].

The CAP is a decision-making model that guides the assessment of individuals' competency to live independently and manage their finances (Figure 1). It is important to note that the CAP presents a dynamic approach because it can include interventions specific to each of the five steps. In addition, the model allows previous steps to be repeated if a reassessment is needed following an intervention or if an assessment requires further enquiry. Thus, the CAP gives health and social service professionals considerable leeway with respect to how they assess an individual and which interventions they make. For example, they may continue their inquiries until they obtain the information needed to initiate the ethical reflection that will enable them to make an appropriate recommendation.

The variables to be assessed in CAP steps were identified through focus groups and by analyzing professional development workshops available to health and social service professionals. These variables were then included in the CAT, the assessment tool, and operationalized version of the CAP. The CAT therefore details the variables used to assess adults' ability to take care of themselves and their property. Health and social service professionals are not required to assess all of the proposed variables in this tool; they should target those that relate to the person's current situation in order to make an informed decision about the applicable risks. The following section describes each step of the CAP and outlines the variables included in the CAT.

### 3.1. Description of the Variables

**Step 1 (causes related to competency assessment).**

A Competency Assessment Process is triggered by difficulties encountered by the adult in functioning independently and safely. The reasons for the assessment may be many and be varied.

The first aspects evaluated are the person's medical condition (physical and mental health), as well as their medication and cognitive functioning. Other elements are examined, including the person's medical history, functioning prior to the evaluation and psychosocial history. The information obtained chronicles the changes that have occurred in a person's condition that warrant the competency assessment. Finally, habits that may affect the person's safety (such as dependence on tobacco, alcohol, drugs, or gaming) are explored.

**Step 2 (functional assessment).**

It is recognized that although cognitive impairments are usually at the root of competency assessment requests, the interdisciplinary team performing the assessment must remain cautious and avoid concluding that a person is incompetent solely on the basis of these impairments [23]. In addition, because clinical assessments of competency must illustrate the need for protection, it is mainly the impact of cognitive impairment on daily functioning and the associated risks that must be well documented, as this will indicate if the vulnerable person needs to be protected or not.

Thus, adults' functional capacities must be evaluated to determine if the person can perform the daily tasks required by their living environment independently and safely. The tasks observed must be related to the aspect being assessed (taking care of oneself or of one's property). It is important to know how the person functioned previously because “marginal” habits, such as keeping many animals in the home, could be misinterpreted if this is a long-standing habit and not a new behaviour. In regard to the ability to manage one's finances, Marson [24] identified nine basic financial skills that could be used for evaluation. These were integrated in the CAT.

To assess changes in habits and to correctly determine if these are signs of incompetency, one must possess information about the person's previous capacities and living environment. Therefore, it is essential to assess people in their own environment or one similar to their own.

To complete this step, variables pertaining to individuals' perception of their capacities and need for assistance, knowledge of their situation and functioning, and ability to protect themselves were added to the CAT [7].

**Step 3 (systemic assessment of the person and their environment).**

This systemic assessment gives professionals the opportunity to determine if people have the capacity to perform their tasks and fulfill their roles in their living environment. Various elements must be documented, including living conditions and available resources, such as services close to home. Environmental (e.g., residential location) and physical (e.g., living on an upper floor of an apartment building) requirements and human needs (e.g., having a dependent) are also included in the assessment.

The presence or absence of aggravating and attenuating factors, such as social isolation and abusive situations, must be documented. For example, an isolated senior may be at greater risk than one with family support. Other elements to examine are the person's actual conditions (assets, housing, financial situation, food, etc.) as well as available personal, community, and private resources.

Documenting the desires, values and beliefs of the adult and his/her family can expand the systemic assessment. According to the 1990* Public Curator Act* in Québec, people have the right to decide for themselves. Since professionals' ultimate recommendation will have a major impact on individuals and their rights, it is essential that the person's preferences, spirituality, and values are taken into consideration [25] and included in the ethical reflection in Step 5.

**Step 4 (situation analysis and risk identification).**

Once the first three steps are documented, the real risks the person faces are identified and rated on a scale from low to high risk. The problems identified at the beginning of phase 1 are analyzed and a degree of risk is estimated for each one. After estimating the risk, consideration must be given to the presence of aggravating and alleviating factors that may influence the risk facing the person being assessed. For example, moderate dementia may represent a low risk for a person living with a family member, but a high risk for a person living with a dependent person.

**Step 5 (ethical reflections and decision-making in interdisciplinary team).**

Ethical reflections require carrying out in-depth research on the values and ethical issues involved with the assessment of adults with mental illness or cognitive impairments. At this step, the health and social service professional reviews the essential elements gathered in the previous steps (significant impairments, risks, aggravating, and alleviating factors) and decides on various options for the person. The positive and negative aspects of each option are analyzed, with consideration given to personal, organizational, and professional values, as well as to ethical issues identified by the interdisciplinary team.

Discussions between team members are guided by a list of specific questions. These questions are a part of a process designed to develop discernment and support and to enrich ethical reflection. Key questions aim to open discussion on the person's and his or her family's values, risks, anticipated positive, and negative consequences and to identify the most acceptable recommendation in the situation.

These questions help to comprehensively analyze the decision and take the various options into account. Consideration must be given to the individual's and family's desires, beliefs, and values; to the codes of ethics of the various health and social service professionals involved; and to the rules and laws in effect in the particular environment.

## 4. Validation of the CAP and CAT

Many steps led to the development and validation of the Competency Assessment Process (CAP) model and the resulting detailed Competency Assessment Tool (CAT). To ensure scientific rigor, triangulation was used when developing the model, that is, theories, methods, and researchers.

The different models found in the scientific literature were first compared to develop the CAP, the theoretical framework underlying the CAT. This decision-making model was then validated with health and social service professionals, including occupational therapists, physicians, neuropsychologists, nurses, and social workers [14].

Next, triangulation of methods was used to catalog the variables that were to be included in the CAT, that is, analysis of professional development workshops available to Québec health and social service professionals since 2005, analysis of data from the scientific literature, and input from focus groups of Québec experts (i.e., health and social service professionals authorized to assess competency). The methodology used in this step is detailed in an article by Giroux and colleagues [21].

In addition to determining some of the variables, the results from the focus groups pointed to the need to add an ethical component to the model. The ethical reflection step, which was added when developing the CAP, consists of questions based on the works of Legault [26] and of Bolly and Grandjean [27]. The questions are designed to guide health and social service professionals during interdisciplinary team deliberations as well as to foster ethical reflection and dialogue within the team.

Finally, to validate the CAP and CAT, observations from researchers were triangulated. The research team who developed both model and tool consisted of three occupational therapists with differing areas of expertise as well as an ethicist. During the process, the researchers consulted experts to revalidate the CAT. The experts commented on the organization of the CAP as well as on the relevance and completeness of the variables used to operationalize it in the CAT.

The CAP and CAT were developed in Québec, which is largely French speaking. For publication and knowledge transfer, a certified translator translated the model and tool into English. Then, two English-speaking occupational therapists validated the English versions of the CAP and CAT.

The study was approved by the Laval University Research Ethics Committee (2007-133 R-1). The experiment was conducted with the consent of the health and social service professionals who participated in the study. These professionals received financial compensation for their expenses.

## 5. Application Study of the CAP

### 5.1. Method

Over the past two years, two professional development workshops designed for health and social service professionals used the CAP as a theoretical model to guide clinical competency assessment. However, the application rate of the CAP in clinical practice following the training and the impact of its use have not yet been documented. The objective of this study was to document the rate of application of the CAP by health and social service professionals following the training and the impact of its use in clinical practice.

A survey was conducted to collect information on the use of the CAP model by health and social service professionals following the training. A total of 34 health and social service professionals were recruited randomly from all participants who completed this training.  Table 1 shows occupations held by this sample of participants. These were first contacted by email. If they agreed to participate, a link to a web survey hosted on a secure site was emailed to them.

Table 2 shows the questions that were sent to participants via the web survey. The majority of them were closed questions (yes/no), but three were open-ended questions that aimed to collect qualitative data (questions (1b), (2d), and (4)).

Results were compiled and analyzed. Analysis of quantitative data collected from the closed questions was done using percentages since the objective of this study was not to ascertain the effect of a treatment, but rather to describe the use of CAP/CAT. For qualitative questions, the data were compiled and analyzed using the analytic questioning approach proposed by Paille and Muchielli [28].

## 6. Results

From the 34 participants initially recruited, 26 fully completed the web survey and four have partially responded. It appears that 40% of participants are currently using CAP. Of these, nearly 86% said that it helps make a more comprehensive and rigorous assessment of competency. “It helps me create a better structure to highlight the factors I must consider for my evaluation” (P21). Among the comments received, it appears that using CAP allows us (1) not to omit elements; (2) to structure the assessment; (3) to target the issues; and (4) to better understand the considerations for decision-making.

Among the participants who are not currently using the CAP, 61% said that they did not use it because they have not had the opportunity to perform a competency assessment since receiving the training. In addition, 33% of them report using some part of the CAP or using it as a guide, but not using it completely. Finally, a participant noted that the CAP does not apply to his clinical context.

When asked if they used the CAP model for writing reports, 26% of respondents indicated that they currently use CAP to guide them in the writing of their evaluation reports. Up to 45% of those who say they do not presently use it plan to do so eventually. Among these participants, some explain that the main reason they do not currently use the CAP as a report template is that “writing frameworks have already been established in our organisation” (P9).

Nearly 89% of participants who currently use the CAP as a canvas find that it helps to better organize information and facilitates a better understanding of the findings by other team members. One participant mentions “I received very good comments from the people who have read my reports with the CAP. They found them thorough, detailed and very appropriate to assess clinical competency. One doctor found it so complete and clear that he asked to join a copy of my report done with the CAP to his report and to the forms he has to fill out” (P13). The main reason mentioned by participants for using the model is that it allows clearer identification of the risks as well as of the contributing factors to those risks.

Finally, even though the training was not given to every member of the interdisciplinary team, participants report that it had a positive impact on their colleagues. “The social workers told me that they had never received such detailed and comprehensive occupational therapy reports to help them fill out the psychosocial form needed for opening a protective supervision” (P22). This finding highlights the relevance of using the CAP even if the clinical context is not favourable to interdisciplinary team work.

Additionally, nearly 85% of respondents believe that it would be relevant that all members of the interdisciplinary team use the CAP. This would optimize this model's positive effects. The reasons cited for this in the comments received are that the CAP (1) provides a common language, (2) standardizes practice, and (3) allows clearly identifying all aspects of the person's situation while avoiding duplication.

## 7. Discussion

The CAP is a dynamic model that allows a rigorous assessment and gives health and social service professionals a great latitude in targeting the variables to assess. It is also versatile, which means that it can be used in different practice contexts. The CAP reflects actual work environments and provides detailed guidance for the assessment by the interdisciplinary team.

The results of the survey highlight the high level of satisfaction of those who use the CAP to guide competency assessment. It appears that this model allows a more comprehensive and rigorous assessment and it brings into better focus the risks and important factors that need to be taken into account when attending to decision-making issues. In addition, although less than 50% of respondents reported currently using the CAP, the majority of them plan to use it eventually and a large majority assert the relevance of this model.

It must be noted that participants highlighted the relevance of using the CAP, even when not all members of the interdisciplinary team use it. Consequently, even though some practice settings might not be suitable for interdisciplinary work, using CAP remains noticeably relevant and appropriate. But the results clearly show that use of the CAP by the entire interdisciplinary team is highly relevant since this level of use optimises the model's contribution to the decision-making process.

Indeed, the inclusion of an ethical reflection in the CAP is an innovative and important addition to the other model currently being used. This addition of this assessment aspect enhances the careful consideration that is given to each situation and contributes to ensure that the best possible decision is taken for each context. Moreover, ethical reflection is likely to have a long-term impact on professionals' awareness and ethical competency, provided that they incorporate the proposed ethical process into their standard procedure.

## 8. Study Limitations

The survey conducted included health and social service professionals that have received training. It is possible to surmise that the participants were interested in changing their clinical practices prior to this training. This could represent a favourable bias towards the application of CAP. In addition, the study did not solicit all members of the interdisciplinary teams which could limit the reach of the results on the impact of its use since step five of CAP concerns all members of these teams. Further studies are needed to document this impact.

The use of the survey technique limited our capacity to establish if links exist between data gathered and participants' occupation or other characteristics (gender, age, etc.). Thus, it is not possible to determine if participants' characteristics influenced the use that was made of the CAP. Also, some questions applied to only some of the participants, limiting the sample of answers that could be gathered for these queries.

## 9. Ongoing Research

Two studies that aim to continue validation of the CAT are now under way. First, focus groups are being held with older adults, senior advocacy groups, and family caregivers to complete validation of the CAT. Second, a content validity study is also taking place with the collaboration of ten legal experts (lawyers, notaries, and judges) usually involved in determining legal competency.

The CAT tool will initially be tested with a pilot project involving several health and social service institutions. The project will examine its implementation and its use on a computerized medium with a targeted interdisciplinary team working with individuals whose competency is being assessed. Results of this pilot project will provide an evaluation of the methods of knowledge transfer used to support the implementation of the CAT, the identification of the ones that are the most effective, and will ultimately result in a corresponding improvement of professional practices.

## 10. Conclusion

Assessing adult's competency to take care of themselves and their property has major implications for the individuals being assessed and their families because, in the absence of validated assessment methods, there is a serious risk of prejudice when declaring someone incompetent. In today's context, with an aging population and increasing demand for geriatric care, health and social service professionals must be equipped to protect the rights and autonomy of vulnerable seniors.

This study confirmed the relevance of the CAP to standardize competency assessment practices and ensure fair and rigorous assessments. Participants confirmed that the CAP is a feasible and relevant model, even when it is only used by one or several members of an interdisciplinary team. The flexibility of the CAP promotes its use in different clinical settings. In a context where practices are changing, this aspect represents a significant strength of the model. Furthermore, adding a step centered on ethical reflection to the model is a very important contribution. The decision often represents a dilemma for the professionals involved in view of the serious consequences it entails for the person being evaluated. A systematic and thorough consideration of the situation is needed to identify the solutions that are best adapted to each context. This step will provide interdisciplinary teams with a framework for ethical decision-making.

## Figures and Tables

**Table 1:** Sample size according to occupation.

Profession	*N* = 34
Human relations officer	1
Head of the living units department	1
Home care support services manager	1
Occupational therapists (OT)	11
Nurse	5
Neuropsychologist	2
Physical therapist (PT)	1
Social worker (SW)	8

**Table 2:** Questions included in the survey and quantitative answers.

Question	*n*	Choice	Total	%
(1) Since you followed the training on competency assessment, did you use the CAP model to guide your evaluation?	30	Yes	12	40
		No	18	60

(1a) If you answered yes, do you consider that it helps you to make a more detailed and rigorous assessment?	12	Yes	11	91,67
		No	1	8,33

**(1b) If you answered no, can you give the reasons that motivated you not to use the CAP model to guide your assessment?**				

(2) Are you using the CAP model for writing your reports?	27	Yes	7	25,93
		No	20	74,07

(2a) If you answered yes, do you consider that this helps you to draft your reports?	7	Yes	6	85,71
		No	1	14,29

(2b) If you answered yes, do you consider that it helps your colleagues have a better understanding of your findings?	6	Yes	6	100
		No	0	

(2c) If you answered yes, did it have an impact on the use of your reports by the social worker?	7	Yes	5	71,43
		No	2	28,57

**(2d) If you answered no, what is the main reason behind the choice not to use CAP as a report template?**				

(2e) If you answered no, do you plan to use the CAP reporting format eventually?	19	Yes	9	47,37
		No	10	52,63

(3) Do you think it would be appropriate that all team members use the CAP to assess competency?	26	Yes	22	84,62
		No	4	15,38

**(4) What the main challenge you face when assessing competency? **				
